
JOURNAL INFORMATION
==============================
NLM Title Abbreviation: ScientificWorldJournal
Journal ID: ScientificWorldJournal
NLM Journal ID: 101131163
Journal ID: TSWJ

The Scientific World Journal

ISSN: 2356-6140
EISSN: 1537-744X
Publisher: Wiley

ARTICLE INFORMATION
==============================
PMCID: PMC6009344
PMID: 29973795
DOI: 10.1100/tsw.2002.21
Article ID: 507304
Article version: 1
Subjects: Short Report

Probing Single Icam-1/Lfa-1 Interaction under External Force

Zhang Xiaohui *
Wojcikiewicz Ewa
Moy Vincent T.
Department of Physiology and Biophysics, University of Miami School of Medicine, Miami, P.O. Box 016430, Miami, FL 33101, USA
*Xiaohui Zhang: xzhang@newssun.med.miami.com
Publication date: 2002
Electronic publication date: 2002 May 22
Volume: 2
First page: 41
Last page: 42
Copyright: Copyright © 2002 Xiaohui Zhang et al.
Copyright year: 2002
License: This is an open access article distributed under the Creative Commons Attribution License, which permits unrestricted use, distribution, and reproduction in any medium, provided the original work is properly cited.
License URL: https://creativecommons.org/licenses/by/3.0/

==============================
Advances in Gene Technology: The Genome and Beyond –
Structural Biology for Medicine (Proceedings of the 2002 Miami
*Nature Biotechnology* Winter Symposium)
*TheScientificWorld* 2002, 2(S2), 41–42
ISSN 1532-2246; DOI 10.1100/tsw.2002.21

### PROBING SINGLE ICAM-1/LFA-1 INTERACTION UNDER EXTERNAL FORCE

Xiaohui Zhang, Ewa Wojcikiewicz, and Vincent T. Moy
Department of Physiology and Biophysics, University of Miami School of Medicine, Miami, P.O. Box 016430, Miami, FL 33101
xzhang@newssun.med.miami.com

**INTRODUCTION.** The interaction of lymphocyte function associated antigen-1 (LFA-1) with its ligand, intercellular adhesion molecule-1 (ICAM-1), mediates firm adhesion between leukocytes and vascular endothelial cells. Unlike other ligand-receptor systems, leukocyte adhesion molecules are constantly exposed to shear stress and pulsatile forces in the blood stream. The likelihood of getting firm adhesion is largely dependent on the dissociation/unbinding properties of adhesion molecule complexes under external pooling force. These properties cannot be obtained from conventional rate constant measurements. Herein we introduced Atomic Force Microscope (AFM) to measure the dissociation properties of single ICAM-1/LFA-1 complex under external pooling force. A similar AFM system has been used to study biotin-avidin interaction[1,2].

**METHOD.** AFM force measurements were carried out at 25°C using a home-built AFM[2] in RPMI 1640 medium. 3A9 cells (a murine T-cell hybridoma) were attached to the AFM cantilever by Con A-mediated linkages. A truncated form of murine ICAM-1 (sICAM-1)[3] was physioadsorbed overnight at 4°C on 35-mm tissue culture dishes. 3A9 cells were first brought into brief contact with ICAM-coated dishes to allow molecules to interact and retracted with a linear increase of pooling force applied on the ICAM-1/LFA-1 complex prior to bond dissociation. To access the strength of the individual LFA-1/ICAM-1 interaction, contact between the cells was minimized by reducing both contact duration (<50 ms) and contact (compression) force. We were able to acquire measurements with force loading rates between 10 and 50,000 pN/s by adjusting the retraction rate of the cantilever from 0.05–15 µm/s.

**RESULTS.** The LFA-1/ICAM-1 bond was stretched by the cantilever until it finally ruptured, resulting in a sharp transition in the force-displacement curve. The measured unbinding force of the complex was derived from the magnitude of this transition. The unbinding of the LFA-1/ICAM-1 complex by the application of external forces was examined in the context of the Bell model[4]. In this model, an applied force $f$ distorts the energy landscape of the LFA-1/ICAM-1 complex resulting in a lowering of the activation barrier(s). The AFM force measurements revealed two activation barriers in the dissociation of the LFA-1/ICAM-1 complex. The outer energy barrier was obtained at the slow loading regime of 10 to 10,000 pN/s. The $k^{0}_{off}$ for the dissociation of the low affinity LFA-1/ICAM-1 complex in this loading regime was 2 $s^{-1}$, whereas the $k^{0}_{off}$ for high affinity LFA-1/ICAM-1 complex was 0.1 $s^{-1}$. Both numbers are in good agreement with published kinetic measurements. The inner energy barrier was obtained in the high force loading regime (>10,000pN/s), which revealed the same $k_{off}^{0}$ (about 35 $s^{-1}$) for both low and high affinity state of LFA-1. $k_{off}^{0}$ for the inner barrier reflected the events (e.g., collapse of a H-bond network) that occur during the initial unbinding of the LFA-1/ICAM-1 complex and is not accessible by conventional kinetic measurements.

**REFERENCES**

1. Moy, V.T., Florin, E.L., and Gaub, H.E. (1994) Science 266, 257–259.
2. Yuan, C., Chen, A., Kolb, P., and Moy, V.T. (2000) Biochemistry 39, 10219–10223.
3. Lollo, B.A., Chan, K.W.H., Hanson, E.M., Moy, V.T., and Brian, A.A. (1993) J. Biol. Chem. 268, 21693–21700.
4. Bell, G.I. (1978) Science 200, 618–627.
5. Evans, E. and Ritchie, K. (1997) Biophys. J. 72, 1541–1555.
